# Supplementary material for: In silico analysis of local RNA secondary structure in influenza virus A, B and C finds evidence of widespread ordered stability but little evidence of significant covariation
Source: Sci Rep. 2022 Jan 10;12:310. doi: 10.1038/s41598-021-03767-x (PMC8748542; doi:10.1038/s41598-021-03767-x)
Supplement: Supplementary file 1 — Supplementary Information. [file 41598_2021_3767_MOESM1_ESM.zip › Supplemental Files/Supplemental File 3.zip!/Supplemental File 3/CM-CAll-rev/pdf/ICV_seg3_2_1.R2R.sto.pdf]

ICV_seg3_2_1

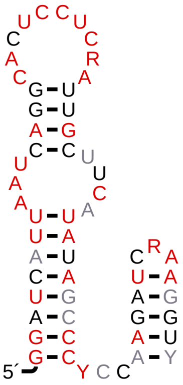
U C C U
C C
A R
C A
G U
G U
A G
C C U
U U
A C
A A
U U
U A
A U
C A
U G
A C
G C
G C
5´
Y C C
C R A
U A
A G
G G
A U
A Y
